# Fifth Annual Meeting of the American Dental Convention

**Published:** 1859-10

**Authors:** 


### PROCEEDINGS OF SOCIETIES.

### ARTICLE X.

*Fifth Annual Meeting of the American Dental Convention.* Reported for the American Journal of Dental Science.

The fifth annual meeting of the American Dental Convention took place in Grant's Hall, Niagara Falls, on the 2d day of August, 1859. The Convention was called to order at 11 o'clock, A. M., by the President, Dr. Isaiah

Forbes, of St. Louis, who cordially welcomed the members to Niagara Falls, suggesting the propriety of making their remarks short and to the point.

The Secretary, Dr. Taft, read the minutes of the last meeting, held at Cincinnati, which were adopted.

The President stated the first order of business to be the report of committees.

Dr. Allport, of Chicago, moved that the miscellaneous business be taken up before the election of officers.

The Treasurer, W. B. Roberts, read his report, which showed an expenditure of $12 04 over receipts; referred to the executive committee.

The President called for the report of the committee "on memorializing Congress on the necessity of appointing dentists in the regular army." The chairman, (Dr. Taylor,) not being ready to report, further time was granted.

The President stated that for a person to become a member of the Convention, it would be necessary to subscribe his name and pay the sum of two dollars.

The following gentleman subscribed their names during the sitting of the convention.

MAINE.—S. B. Straw, Bangor; J. Mason, Saco.

NEW HAMPSHIRE.—Frank Fuller, Portsmouth.

VERMONT.—J. R. Lewis, Burlington; M. Tefft, West Poultney.

MASSACHUSETTS.—J. R. Dillingham, Abel Ball, W. K. Mayo, E. T. Wilson, Boston; W. A. Sanborn, Haverhill; Seth P. Miller, Worcester; Geo. J. Cook, Milford.

CONNECTICUT.—W. Potter, Norwich.

NEW YORK.—K. Kun, Geo. H. Perine, F. H. Norton, C. S. Weeks, T. H. Burras, John Allen, Geo. Clay, E. A. L. Roberts, R. McIlroy, B. W. Franklin, W. B. Roberts, M. Levett, E. Oliver, S. D. Sherman, James Pearson, New York City; L. W. Rogers, C. B. Foster, Utica; C. W. Harvey, L. F. Harvey, John Lewis, Geo. F. Foote, R. G. Snow, B. T. Whitney, Buffalo; J. G. Barber, Leroy; A. Hooper, Binghampton; L. D. Walter, Lockport; C. G.

Sumner, Norwich; J. Naramore, Rochester; J. C. Gifford, Westfield; D. S. Goldy, Oswego; E. M. Skinner, Syracuse; H. A. Coe, Theresa; James Corman, Mecklenburg; E. D. Fuller, Peeksville; H. Hodge, Green, Chenango Co.; S. M. Robinson, Watertown; S. F. Tremain, Rome; E. Elmendorf, Pen Yan; S. N. Smith, Auburn; A. Westcott, Syracuse.

Pennsylvania.—John B. McCurdy, T. L. Buckingham, W. Calvert, Geo. T. Barker, S. S. White, J. H. McQuillan, Philadelphia; A. L. Suesserott, Chambersburg; A. B. Robbins, Meadville; W. H. Luce, Erie, O. L. Elliott, Erie; W. M. Wright, Pittsburg.

Ohio.—W. H. Atkinson, C. R. Butler, G. Langsdorff, B. Strickland, E. P. Crowell, Cleveland; C. M. Kelsey, Mt. Vernon; R. McDowell, Wooster; C. Palmer, Warren, Trumbull Co.; A. A. Blount, Springfield; G. W. Keely, Oxford; A. E. Lyman, Newton Falls; H. A. Smith, S. L. Hamlin, James Taylor, J. Richardson, J. Taft, Charles H. James, J. G. Cameron, Cincinnati; Geo. Watt, Xenia; J. Ramsey, Springfield; C. P. Bailey, Cuyahoga Falls.

Virginia.—E. G. Winchell, Wheeling; John George Wayt, Richmond.

Alabama.—J. S. Lavender, Lafayette.

Florida.—P. P. Louis, Tallahasse.

Georgia.—B. B. Alfred, La Grange; D. S. Chase, Augusta; F. Y. Clark, Savannah.

South Carolina.—Horace Parker, Edgefield C. H.

Missouri.—Edgar Taylor, Palmyra; Isaiah Forbes, St. Louis.

Indiana.—G. A. Wells, John F. Johnston, P. P. Hunt, Indianapolis; Geo. Lupton, Shelbyville.

Illinois.—W. W. Allport, Chicago.

Michigan.—Wm. Cohoan, Detroit; C. B. Porter, Ann Arbor.

Kentucky.—J. L. Nourse, Cloverport.

Wisconsin.—D. W. Perkins, Milwaukee; Isaac George, Kenosha.

New Jersey.—R. V. Jenks, Paterson.

Canada West.—G. V. N. Relyea, Belleville; J. S. Walter, St. Catherines; W. W. White, Chatham, (C. W.)

Vantuyle, Rio de Janeiro, Brazil.

Robert Vandevort. (No town put down.)

The President announced the reception of a communication by Dr. McCurdy, from our trans-atlantic brethren, which he read; it is dated, 15th July, 1859.

No. 5 Cavendish Square, London.

To the Chairman of the Meeting of the "American Dental Convention," to be held at Niagara Falls, on Wednesday, the 3d of August, 1859:

Dear Sir:—We are instructed by the Council of the College of Dentists of England to address you on the occasion of your important annual gathering at Niagara Falls, in the ensuing month of August. A few months since, many of the dentists of this country anticipated the pleasure of inviting their transatlantic brethren to a general professional gathering in London, in the year 1861, at which period, it was understood a universal industrial exhibition, similar to that of 1851, would be held. The hope of such an exhibition at the period indicated, is however dispelled in consequence of the present disturbed state of the continent of Europe, which has not only paralyzed all industrial efforts, but must inevitably retard civilization in many neighboring countries.

You are doubtless aware that differences of opinion in the ranks of our profession in England, have tended very materially to check that unity of action which is so essential to the attainment of perfect organization and success; but we trust that those differences will be speedily removed, so that when the time is opportune for another World's Convention, the *unanimous* invitation of the Dentists of England may go forth to their brethren in America. In this hope the Council most cordially indulge, and it would give to the several members great satisfaction to welcome their American brethren to the shores of Great Britain, and especially with a view to the holding of a meeting in London of the "American Dental Convention."

We have the honor to be, dear sir,

Yours, faithfully,

GEORGE WAITE, M. R. C. S.

*President.*

Samuel Lee Rymer, } *Secretaries.*

Anthony Hockley, }

The communication was received and accepted, and Dr. McCurdy requested to make a proper acknowledgment.

Dr. Taylor, of Cincinnati, moved that each member be confined to ten minutes in the discussions, as heretofore; adopted.

Dr. Allport suggested that some member should be appointed to make a report of the proceedings of the Convention, to ensure completeness and accuracy, and that the dental journals be furnished copies, to use as much as they choose of it.

He moved that Dr. Watt be employed, at — dollars, to make such report. (Dr. A. stated, in explanation, that it was intended to have Dr. Watt employ a stenographer, and revise the manuscript.)

Dr. Westcott remarked that there were reporters present for journals, and that it did not seem advisable for the Convention to hire one on their own account.

Dr. Watt thought it unadvisable to employ a reporter.

Dr. Butler offered an amendment, that a committee of three be appointed with Dr. Watt, to prepare report for publication, &c. Not seconded.

Dr. F. Fuller thought that a better way would be, as there were several reporters present from newspapers and journals, to let them all make their own reports, that it was in some way a reflection on them, and the effect would be to throw these men out of employment. If we have a reporter, let us publish it as the preceedings of this Convention; we are not connected with journals.

Dr. Barker said, that as far as his journal, the *Cosmos*, was concerned, they should only publish a condensed report.

Dr. Gillingham hoped, by all means, the Convention would employ a competent reporter, and have a committee to supervise his work, that it may be full and correct.

Mr. Norton said he did not intend to make a full report for his journal; was reporting as much for a daily paper as for it.

Dr. Allen did not wish any action to be taken to exclude the reporters that are present, but thought the best course would be to have a verbatim report for the use of members. Knew reporters were present that could be had, who would report every word; did not think they could report too much.

Dr. Colgate moved that the whole subject be laid on the table.

Dr. Allport made an explanation, that it was not the intention of his resolution to exclude any reporters, but wished the Convention to have a report of their own.

Dr. Rogers remarked that he had been written to in relation to reporting the proceedings, but had not felt authorized to engage a reporter, though the President, whom he had written, thought it advisable.

Dr. Straw wanted to know the business before the Convention, and called for the reading of the resolution.

Dr. Allport asked permission to withdraw the resolution. Granted.

Dr. Dillingham moved, that this Convention employ a reporter, whose report shall be published in pamphlet form.

Dr. Westcott did not think the Convention would be warranted in employing a reporter, as several were now here, and in the present state of the finances, did not think it should be done with the funds of the Convention.

Dr. Taylor thought a good report desirable; the reporters present are an obstacle to the employing of one by the

Convention. Thought a verbatim report unnecessary, as much was said that was unimportant, and that had better be left out than be put in print; would trust to the good sense of the reporters and the editors of journals. Did not wish to get up any envious feelings between the different journals.

Dr. Dillingham again urged the Convention to secure a competent reporter, and have their doings published; if the American Dental Convention is not able to have a report of their proceedings published, they had better dissolve. That the Convention is second to none in the world, &c.

Dr. Foote remarked, that if the resolution was much further discussed there would be nothing to report.

The question was taken and lost.

The Convention then proceeded to the election of officers, Drs. Fuller and McKellops acting as tellers.

The following officers were elected:

Fifty-eight votes were cast for *President*, of which S. W. Rogers, of Utica, N. Y., received thirty-six, and was declared duly elected.

*Vice-President.*—George Watt, Xenia, O.

*Recording Secretary.*—Frank Fuller, Portsmouth, N. H.

*Corresponding Secretary.*—P. P. Lewis, Tallahasse, Fa.

*Treasurer.*—D. S. Chase, Augusta, Georgia.

The following Executive Committee were elected, *viva voce:*

Drs. G. F. Foote, Buffalo; T. H. Burras, New York City; J. Mason, Saco, Me.; S. M. Robinson, Watertown, N. Y.; James Taylor, Cincinnati.

On motion, it was resolved, that there be but one session of the Convention per day, and that the hours be from 9, A. M., to 2½ P. M.

Adjourned.

---

Wednesday, August 3d.—Second day's proceedings.

Members assembled at 9, A. M.—The President announced the first order of business, after reading the minutes, to be the report of the executive committee on Treasurer's report. The committee reported that they had examined the accounts and vouchers of the Treasurer, and find an excess of expenditures over receipts of $12 04. Report accepted. The President, Dr. Forbes, then addressed the Convention.

Drs. Taylor and Westcott were appointed to conduct the newly elected President to the chair. On being introduced, Dr. Rogers addressed the Convention as follows:

It may not become me to dispute the wisdom of the choice you have made for President, yet a single glance over this Convention assures me that whatever considerations of ability or fitness may have determined your choice, it cannot have been made with primary regard to professional standing or attainments; and while I fear I may not equal your expectations even as a presiding officer, I am yet profoundly grateful for such an expression of your confidence and esteem.

Gentlemen, I am proud of the American Dental Convention. I have attended every session since it was organized, and have always been highly gratified with the personal appearance, general intelligence, liberal views, and moral habits of those who have been present; and I will venture to say, that for courtesy and gentlemanly deportment in their debates, for sobriety and freedom from all excess in their seasons of festivity, these gatherings will have a favorable comparison with general conventions of any profession, trade, or calling in the country. Such has been their character in the past, what shall it be in the future? It seems to be almost impossible to organize an association, even for religious and moral purposes that will not soon be rent asunder by dissensions. If we would avoid such a result, we must shun the rock on which others have split. Let us continue to cherish a spirit of kind concession and fraternal regard while we freely discuss all questions of general interest, let us carefully avoid whatever is merely personal and local. We must strictly adhere to the rule, that as a body, we will not assume to decide upon the merits of any theory, invention, or mode of practice; but leave every member free to pursue his own course, responsible only to his patients and the community in which he lives. And, gentlemen, we must be willing to sacrifice every thing of personal opinion for the sake of harmony; and in all that pertains to the organization and government of this body, submit cheerfully to the will of the majority. Union and harmony under any organization, is better than dissension and division; "united we stand, but divided we fall." By strictly observing the same general policy which has guided us in the past, this Convention will become a permanent and useful institution, an honor to the profession, and a model for other associations.

Thanking you once more for the honor you have done me, I assume the duties of the office, relying upon your co-operation to enable me to discharge them satisfactorily.

The President announced the next subject for discussion to be "When, and under what circumstances should teeth be extracted to procure a correct development of the permanent teeth?"

Dr. Perine suggested that as several essays were prepared, they be read by States.

The President here remarked, that any one having specimens, teeth, or instruments, for exhibition, are requested to place them in the room in the rear of the chair.

Dr. Taylor then read an essay. (See page 526.)

(At this point the President said it had been proposed to have a supper; and the proprietor of the International had proposed to get one up if a sufficient number of names were procured.)

Dr. Ambler said the subject just discussed in the paper of Dr. Taylor, was of great importance; dentists often conflict, and harm is often done by not thoroughly understanding the subject. Was not present at the last meeting, and did not know the order of the discussions; was not prepared to make many remarks, but desired others to enter fully into the subject.

Dr. Westcott asked an explanation of the word "development," whether it was intended to refer to both regularity and the perfection of the permanent set.

Dr. Allport explained that it was intended to embrace both development and regularity—perfect dentition.

Dr. Westcott moved that the subject be divided, and that the discussion be first, relating to the irregularity of the teeth.

Dr. McQuillan thought a better course would be to take up the "development" first.

The quession was divided, and the subject of "the irregularity of the teeth" taken up. No remarks being made for a few moments,

Dr. Bristol remarked, that it appeared, from no one rising to discuss the question, as if the members thought Dr. Taylor's paper had covered the whole ground. For himself, he was well satisfied with them, so far as his experience went. That if those remarks were fully understood, he had grasped the whole subject; he would wish to pass to some other question.

Dr. Taft did not think members understood the original question, and asked to have it read again.

Dr. Fuller, of Peaksville, considered this matter a very important one, and thought the Convention, as dentists, should eliminate the subject, as medical men do not touch on it, having left it in their hands, and hoped it would be discussed in its broadest sense.

Dr. Westcott did not understand that they were confined in their remarks, by the resolution. To practical men, solicitous for the welfare of their little patients, no subject is so important. In his practice he is often called on to extract teeth before the proper time has arrived, sometimes years before the period indicated in the books. Differs from Dr. Taylor. Should the deciduous teeth ever be extracted before nature indicates a necessity for their removal? Seemed to think they should not. Did not wonder that gentlemen felt reluctant to discuss the subject without having cases presented.

Dr. Perkins stated a case. A patient came to him with the two central incisors making their appearance on the lingual side of the temporary incisors, and occupying about one and a half times more space than that occupied by the temporary incisors. They were situated with their front approximal edges pointing forward against the necks of the temporary teeth, the lateral edges presenting backward so that the cutting edges of the teeth occupied a position of about 45 degrees. In such a case he had extracted the lateral incisors.

Dr. Perkins stated another case, and asked Dr. Westcott what should be done. To which Dr. W. replied, that begging the gentleman's pardon, he did not come here to counsel, (laughter,) and at the suggestion of the President he answered his own question.

Dr. Whitney said that Dr. Perkin's case called to mind one precisely, or very nearly the same, that he had treated quite differently. The superior central incisors appeared on the lingual side of the deciduous ones, the front edges pointing forwards, and the lateral backwards; the cutting edges standing nearly at right angles. I extracted the two deciduous teeth, and left the case for further observation, giving directions to make pressure upon the advancing teeth to aid in bringing them into place. But they soon became interlocked with the under teeth. I inserted a plate, and brought them into their places, or so far as to overlap the under teeth. The patient has now reached her sixteenth year. The arch is somewhat contracted, or rather I should say not rotund, but has the conformation of her family. The front teeth are in good position, except somewhat crowded. I have extracted during the last year the first bicuspid on one side, and the first molar on the other, these having become carious. In Dr. Perkin's case, where will he stop extracting? As one tooth after another advances, it will occupy the place of its next neighbor, I never extract a temporary tooth to make room for a permanent one, other than the one it is to replace: believing that too early professional interference will often do more harm than good; and that the presence of all these teeth are importaut aids in the full development of the alveolar arch. There is a class of deciduous teeth—the molars—that we are daily called upon to extract, to cure or prevent pain; the parents, and I am sorry to say, some dentists, thinking that as they are but temporary teeth, it is of little consequence when they are removed. I never extract them unless ulceration or a badly diseased state of the roots or soft parts demand it; believing it better for the patient to suffer an occasional pain, and resort to palliatives, than to resort to the forceps.

Dr. Allen understood the object of the meeting to be to state their own views and mode of operating; that his advice is to have the temporary molar teeth retained as long as possible. The dentist should remember that the jaw is constantly growing. If the temporary teeth are sound, by all means retain them; has seen many cases where the canine and incisors were close together.

Dr. Cormack, of Scaylee, thought the question had arrived just at this point. If irregularity occurs it is hereditary—related a case, that of a young man who had several incisors emerging like Dr. Perkin's patient; the lateral incisors were extracted, the front allowed to grow. They have waited twenty-five years for the appearance of the lateral incisors. The deciduous central incisors remain and are filled. If they had been extractcd he would have been in the same situation of his great uncle. Thought from cases cited, we could not fix upon any time for the extraction of the deciduous teeth.

Dr. Sanborn has found in his practice, that the lateral deciduous do not give room for the permanent incisors. The laterals are perhaps firm, and would remain no longer than it would be judicious to leave them in place; is often perplexed to decide when to extract the lateral incisors. Inquired if it was practical to leave the deciduous teeth until absorption took place.

Dr. Asay. When children are brought to him, keeps his eye on two or three matters. Is much guided in his treatment if he can see the parents. If the child has a crowded arch, first took into consideration the means to correct that. If the parents have contracted arch, the children are more likely to, as children are much like their parents. Related the case of his own son who had a contracted arch, resembling his own; who, at seven years of age had not shed either of the diciduous molars; after these were removed there was an inclination to point backward. Did not think it advisable to remove the deciduous until the permanent teeth appear.

Dr. Straw did not think the deciduous teeth, if decayed, worth filling, would extract them to give room to others.

Dr. Clay. It has been my practice to leave the first teeth as long as possible; I had a boy who had ulcerated teeth at eleven months, and I extracted them, and was fearful he would not have the second, but they appeared at last, and the teeth were well set.

Dr. Coane, of New York, wanted to know if it was a fact, that the temporary molars were more liable to ulceration than the temporary incisors. Stated a case where he had extracted a fang and reached a perfectly formed incisor. Asked if the premature extraction of the primary teeth did not retard the appearance of the permanent set.

The Chair thought too much time was being taken up with the subject, as there were many other topics to discuss, when it was resolved that the Convention take up the second division of the subject, "The development of the teeth from the rudiments."

Dr. Atkinson thought much time was consumed to no purpose—hardly considered this question of profit for the Convention, as the dentist has little or nothing to do with the development of the organization. As to the contraction of bone spoken of by some, it was impossible that the jaw should contract. It should be stated as a non-development. He would save the temporary teeth even when dead, would run a risk if at all, on the safe side.

Dr. McQuillan has always been convinced that cause precedes effects. We should first consider how the fangs are absorbed. The ancients say they have no fangs ; later, that the absorption is due to an acid. Another theory is, that the pressure of the permanent tooth produces the absorption. Delabarre says there was a carneous, spongy body, whose office it was to produce absorption of the temporary teeth. Thought none of these three theories correct, we must go back to disintegration and deposition. We have in the body various glands, as the thymus, thyroid, &c., these are absorbed until ultimately only a trace remains. Why may we not say the same thing occurs in the case of the temporary teeth. The teeth, and the various glands, and parts of the body derive from the blood such nutriment as they need, and as the body presents no cases of glandular absorption, why may we not apply the same theory to the first organs in the mouth. In relation to the extraction of teeth, he would wait till there was a permanent tooth below, before he would extract the temporary.

Dr. Taft said he thought the first part of the discussion took a wrong course. Instead of taking cases which may all have peculiarities, the discussion should be confined to general principles. If the temporary teeth are not absorbed at the right time, and the permanent appear, then we should extract the temporary ; but if the temporary are not absorbed, and the permanent do not appear, then the temporary should not be extracted, unless there were also present disease that would be pernicious. We have cases where the permanent do not appear, and the temporary teeth remain and are good. What effect the temporary had on the permanent teeth he did not know, but wished it might be thoroughly discussed.

Dr. Miller understood the question to be, what influence the temporary have on the permanent teeth, and asked, if we are to remove the temporary. Often have cases presented where there is imperfect absorption of the dead tooth. Spoke of the shape of gums, and remarked, if there were any present with rotund jaws, they should do all they could to hand them down to posterity.

Dr. Westcott had a case where the canine tooth of the permanent set appeared one and a half inches from where it ought to. Has extracted the cuspids as late as the twentieth year, and had its successor appear. Did not think any could explain the process of absorption of the temporary; perhaps that of Dr. McQuillan was as plausible as any. Aside from the diseased state of the roots, regarded the first teeth as having little effect on the permanent.

Dr. McQuillan thought there was a decided misapplication of the words "development" and "growth," and gave Paget's explanation of the terms.

Dr. Roberts, of N. Y., related a case where the permanent teeth came in only at the thirty-fifth year.

Dr. Whitney said the remarks made in regard to the presence or absence of the temporary teeth having an influence on the development of the permanent ones, called to mind a case of some interest on this subject. A little girl, about three or four years old, (as near as I can recollect,) fell upon stone door steps and drove the four superior incisors and one cuspidati, upward, splitting off the alveolar process; the cutting edges of the teeth being buried under the gum over the alveolar ridge. I removed the teeth, fearing at the time the germs of the second teeth might be injured. I waited for a number of years after the usual time for the second teeth to appear. I lost sight of the patient by removal, and do not know whether she had a dental adviser during this period or not. She is now about eighteen years old, the permanent teeth occupying the place of those that were knocked out, are perfect and nearly in their proper position, though very much crowded. The arch is not as well developed, as she is constitutionally entitled to, judging from the full rotund arch of the mother, and tolerable development in the father, and a good development in the two other children. I am led to believe that the presence or absence of the temporary teeth has no influence upon the development or growth of the permanent ones, but may have upon their eruption, and certainly do have upon their regularity, as well as the full development of the dental arch. In regard to Dr. Westcott's remarks about the determination of the second teeth to grow or to be fully developed, whether they are allowed to occupy their proper places or not, and citing cases in illustration, calls to mind a case of an opposite nature. Some twelve or fifteen years ago, a gentleman some twenty-six or eight years old, presented himself for a partial set of upper teeth. Some of the teeth had previously been extracted. I removed the remaining fragments and the defective teeth. About a year after this he called to consult me about a little protuberance that had recently appeared in nearly the centre of the dental arch, and was gradually increasing; finally the point of a tooth made its appearance. I removed one of the artificial central incisors, as that was its location, and cut a hole through the plate; it was of very slow growth, but finally it became in appearance a pretty well developed canine tooth, and the last I knew of him, some years after, it was sound and healthy. Now was this a supernumerary or a superior cuspidati, that had been prevented from being developed by its confined position among the roots of the other teeth? I think it was one of the canine teeth of the second set, but more modest than Dr. W's determined ones.

On motion of Dr. Westcott, it was resolved, that no person shall speak but once upon any subject until all have had an opportunity.

The Convention then proceeded to the next subject—"Mechanical appliances and forces to correct irregularities of the teeth."

Dr. Westcott remarked, that one of the greatest difficulties of the dentist was in correcting a spreading of the teeth or jaw. The instruments for this and all other purposes about the teeth for regulating their position, should be of the most simple kind, that the patients themselves can manage them. Showed a model and explained how he had managed some cases.

Dr. W. B. Roberts remarked that the most common occurrence of these malformations was in the young, who had little patience; that in regulating the teeth, the simplest fixtures should be applied. He used a vulcanite plate nicely fitted to the mouth, this is worn with comfort. Where a tooth was to be moved out, or the jaw expanded, this was accomplished by making a standard by the tooth to be removed, saw a thin slit in it, and push a wedge of pine wood in, renewing this from time to time. The vulcanite was also used for making inclined planes for the under jaw; it being so easily worked it was useful in most all cases of irregularity.

Dr. Buckingham had treated some cases like those reported by Dr. W., in a different manner, which he detailed.

Dr. Straw inquired if the spiral springs did not produce irritation? Dr. B. thought they did.

Dr. Franklin said, from his own experience, he had found the vulcanite base to be decidedly the best substance, and gave the names of a number of prominent members of the dental profession who used it exclusively.

Dr. Asay thought it important that the apparatus for regulating the teeth should be worn day and night, and so secured that they may not get into the throat of the patient; had found dry willow the best substance for wedges, as it expands more than anything else.

Dr. Wescott does not commence to regulate the teeth until the first are shed. We need not be afraid to attempt the regulation of the teeth because the patient is twenty or more years old; related a case where they had been regulated at an advanced age. Does not like rubber, it does not know when to stop expanding. Puts in pine wedges, letting them remain two or three days. Does not think spiral springs should be used, they are apt to excite inflammation. When he was young, he had fought processes, but he had got over that, and now does not care what the process is, if it is good and accomplishes the purpose.

Dr. Atkinson asked if it was advisable to bring down a tooth when it was completely developed, and is not on a level with the others?

Dr. Westcott replied, that he would not apply force to bring teeth to a level with others, would wait for nature to apply the force below at the fangs.

Dr. Atkinson remarked, his practice was different, would apply force to bring teeth fully developed on a level, and related a case where he had brought a canine down by traction.

Dr. Ambler related a case of irregularity, occurring in a young lady. When the Convention adjourned.

---

Third day.—Convention assembled at 9, A. M.

Dr. Forbes moved, that Dr. Solyman Brown be permitted to make a statement in relation to a work he contemplates issuing; and on motion of Dr. Taft, 11 o'clock was agreed on as the time for his remarks.

The Convention, on motion, then proceeded to take up the next subject on the programme, viz. "Under what circumstances, and in what manner, should teeth be filed to remove superficial decay, or preparatory to filling, to leave them the most secure from future decay."

Dr. Taft, on certain classes of decay, and in certain positions, he would use the file; would use it on the approximate portions of the teeth, more particularly on the anterior than the posterior portion. If it is superficial decay, on firm, well constituted teeth, he would remove all of it, leaving no concavities. There are cases in firm, strong teeth, where cavities are left and yet no further progress will be made in the decay. Take a soft tooth and cut out and leave a depression, and decay will go on and progress rapidly. A firm, strong tooth may be treated freely with the file, but we cannot leave shoulders or irregularities in frail teeth without danger. The filed surface should always be highly polished. When the file is used, the tooth should be well supported. Can use cutting instruments better, on sensitive dentine, than the file, as every one knows the use of the latter is almost intolerable.

Dr. Atkinson was in favor of the use of the file, and stated that early in his practice the file was used more than now ; the teeth being often filed down even to pegs, these same teeth are now good.

President Rogers asked how far the enamel preserved the teeth? Thought that the bone, if well polished, would resist decay as well as the enamel.

Dr. Chase thought the file one of the most useful instruments in the hands of dentists at the south, and advocates free filing. Thought the openings between the teeth should be largest on the lingual side. Did not fully agree that the teeth should be left on a plane, but would leave them move as nature does, on an oval. Leaves a shoulder; if the surfaces are left plane, decay begins, but if a shoulder is left, they are easily kept clean. Thinks the beveled file the best to finish with.

Dr. Perine thought the file too much used, too harshly. All are not sufficiently careful to select proper cases for its use. The temperament and constitution of the patient should be taken into consideration. There are many that the file may be used freely on. Thought the beauty of the teeth often marred by its use. Alluded to tribes who file the teeth to a point and decay does not appear ; but this should not encourage the student to operate on the teeth of Americans.

Dr. Fuller (Peekskill) alluded to tribes in Abyssinia who file the teeth to the bone, and yet they do not decay. He uses the oxyd of tin, the same used by marble workers, to polish the teeth; using it on buckskin, or with a smooth stick. Considers the error was on the too little use of the file. Experience had taught him that removing the decay well and thoroughly, polishing was the best. It is his plan to file the lingual side most, as by having the largest opening inward, there was more tendency to free the teeth. Hardly ever leaves shoulders to his teeth.

Dr. Westcott thought the subject should be divided into prevention and preparatory, and would confine his remarks to the latter part of the subject. The matter of filing depended very much on the age of the patient. In children, all know that the pulp cavity is sooner reached than in adults; uses the file little on children. His practice is to wedge the teeth of the young—spreads the teeth in order to give room for the file; uses wooden wedges, and prepares for filing as well as filling. The practice of wedging answers well for ladies when it is desirable to preserve a beautiful set of teeth. With careless people, who do not cleanse their teeth, is in the habit of filing freely, leaving open spaces. In filing between the molars, it is his custom to leave a V shaped opening, pointing it in or out, as the cavity is nearest to either side with the front teeth; is in the habit of leaving the upper part of V pointing downwards.

Dr. Taylor agreed generally with the two last speakers; that sometimes there is great recuperative power to withstand decay, and was in the practice of filing sometimes to the extent of one-third of the tooth. Uses a half-round file, thinks it gives the best shape to the molars.

Dr. Forbes never has filed, does not file, and never expects to file for superficial caries. Agreed generally so well with Dr Westcott, that anything he might say about filing would be only a repetition of his remarks. Many years ago, when a student, the ladies were in the habit of having their teeth filed to improve their appearance, and what he had then seen had influenced him against the practice.

Dr. Roberts follows the principle, that when we are in health we need no medicine; that when a tooth commences decaying it did not need medicine. Prefers to wedge the teeth of the young apart, might trim the teeth delicately. Had rather, in those of eighteen to twenty years, run the risk of the teeth coming together, and then filling them.

The President announced that a distinguished literary gentleman, and correspondent of the London *Times*, C. Edwards Lester, was present, and invited him to a seat on the platform.

Dr. Wilson had his teeth filed for superficiad decay and they had been preserved nearly thirty years. He wished to know if Dr. Forbes never uses the file, and why?

Dr. Forbes replied that he was guided by experience. He leaves the decay to progress to a certain extent, when he cleans the cavity and fills it.

Dr. Weeks uses the file for superficial decay, when it has not extended beyond the enamel. When it has, he agreed with Dr. Forbes, that it should be remedied by filling. Uses the file, and polishes with scotch or rotten stone. Early in his practice avoided filing and the teeth turned out bad; uses the file oftener now, and thinks he has saved the teeth more effectually. Does not use the file in the young.

Dr. Allen regarded the file as one of the most valuable instruments in dentistry, but it required care in its use. For superficial caries he uses the file, and burnishes well; thought it better to remove the cause. Could refer to cases where the teeth had been preserved twenty-five years by the use of the file. He gave the case of Dr. Somerby, who had two front teeth filed for superficial decay when a boy, which arrested its further advance, and his teeth were preserved till death, forty years or more after.

Dr. Vandervort had found it more beneficial to file black caries, if it was properly removed it was not apt to return. The yellow or white caries was not so easily removed.

Dr. Perkins stated the case of a patient with decayed spots on the teeth—he removed the surface and polished it and it was perfectly sound years after. He thought when the decay was disfiguring the teeth they should be dressed down and highly polished. Has found that highly polished bone resists decay longer than enamel.

Dr. Ambler thought the abuse of the file was what had created a prejudice against its use. His own teeth were monuments in its favor—his incisors were filed by his father, three or four times, when he was sixteen years old, using a coarse file; they are now good teeth. Was in direct antagonism to Dr. Forbes, as he did now and always expected to use the file as long as anything could be saved by it. Thought it bad argument to say we will not remove decay when superficial, but wait till it has made progress sufficient to fill.

Dr. Forbes remarked that he had the honor to have been a student of Dr. Ambler's, and as the Dr. had shown his teeth as monuments in favor of the use of the file, he desired to show his as monuments against the success of the file. This created considerable merriment—so much interest being shown in this discussion.

Dr. Westcott moved that a vote be taken to decide what proportion of the Convention would file the approximate surfaces of the teeth, at the present day, for superficial decay.

A number of members came to their feet, some in favor of the division, and others stating that they would have to vote on both sides of the question. The question was finally put, and it was decided, carried by the filers; though it was very evident neither party were satisfied. (Dr. Brown [of N. Y.] now made some remarks on a History of Dentistry and a Biography of Dentists, which he proposes issuing during the following year.)

Dr. Allen moved the suspension of the rules for the purpose of introducing a resolution. Carried.

*Resolved,* That this Convention approves of the professional work contemplated by Dr. Solyman Brown, called a "Dictionary of Dental Biography," and recommend to the dental profession in the United States its hearty and liberal encouragement.

Dr. A. proceeded to speak of the merits of the work, and the capability of the author for his task.

The President, Dr. Rogers, was opposed to the resolution, as it conflicted with the course heretofore pursued by the Convention, and it would establish a precedent that would be fatal to the best interests of the Convention.

The question was further discussed by Drs. Asay, Straw and Kelsey, in favor, and Drs. Taylor, Allport and others opposed; when on motion, Dr. Allen withdrew the resolution and offered the following:

*Resolved,* That this Convention are glad to learn that Dr. Brown contemplates the publication of a work to be called "The Dictionary of Dental Biographies." Carried.

All thought the work should be encouraged, but the majority were opposed to committing the Convention for or against anything of the kind. This, with all other works or instruments, should be legitimately advertised and stand upon its merits.

The President announced the next question for discussion to be "Filling Teeth."

Dr. Perine opened the debate, and spoke of the difficulty often experienced in confining the flow from the parotid ducts, and showed an ingenious contrivance of Dr. Hawes' of N. Y., to prevent it. It is of silver, so arranged that it may be adapted to any length of chin. Dr. Hawes offers the benefits of the contrivance to the profession.

Dr. Ambler stated his practice, where cheapness was required in filling, to be as follows: after removing the decay from the fang as much as possible, forces in a gold plug to the extreme point of the fang, has then filled the balance of the fang with gutta percha, then put a cap over the gutta percha. Regards gold as the best filling—uses the other where cheapness is required.

Dr. Allport inquired the object of filling in that way.

Dr. A. replied to save time.

Dr. Allport also inquired if Dr. Ambler could not fill entirely with gold, and do it quicker. He replied he could not. His object in using the cap was to get a solid base.

Dr. Allen presented some cotton that was taken from under a plug: reveals the secret of ill success. Thought that accounted for the reason why some practitioners do work so cheap. Nothing is so good as good gold for filling.

Dr. Forbes referred to the remarks of an English journal, that inquires why American dentists stand higher on the continent than the English? He thought it due to the superior education of the masses in this country, or because the dentists have instructed them to demand good work. Then the English dentists do more cheap work, and he contends that making cheap work to day incapacitates the dentist from making good work next day. Fills in the following manner: 1st. Prepares the cavity. 2d. Prepares the gold. 3d. Prepares the mouth. 4th. Filling. 5th. Finishing. A gentleman suggests, 6th, getting pay. The Dr. thinks that follows as a matter of course. Removes all discoloration, breaks away the enamel to sides, and then cleans out the cavity, using as large instruments as the cavity will permit. Makes the outside of the cavities on a bevel, its mouth largest, for two reasons, first, because it makes a head for his filling. Second, because the filling looks larger than it really is. Uses No. 4 gold, preparing it as follows: cuts up a book at a time, book and all. Rolls his unadhesive gold on a broach, prepares his cylinders to fit the size of the cavities. Mouth.—Wipes the gum and lays on a piece of muslin, folding a napkin around the tooth. If he has a cavity 3 lines in diameter and 3 lines in depth, he uses a cylinder 4 lines long, pressing laterally, puts another cylinder of 1½ lines in, continuing to press laterally then condenses, then finishes. Makes his cylinders by laying his ribbons on a cushion and rolling his broach.

Dr. Clark said, some years ago he used adhesive foil, but was not successful; now he uses a beautiful foil made by Dr. D. S. Chase, of Augusta. Living, as he does, in a warm climate, the moisture of the hands is a serious inconvenience, and injures the gold. Showed his way of folding without touching it; which is accomplished by the use of two triangular blocks of ivory, about the length of a sheet of gold, which he exhibited.

Dr. W. B. Roberts presented a case of a central incisor, that had been filled several years with amalgam, nearly one-half of the tooth was broken away; an abscess was formed; the glands of the throat much swollen, and the mouth sore, inflammation extending over nearly all the mucous membrane of the mouth; the breath was very offensive, very much like that produced by salivation. Matter was discharging freely around the fang of the tooth, which had made it exceedingly loose. The patient had suffered three weeks previous to calling on Dr. R., having been told that the difficulty did not proceed from the tooth. The amalgam filling was immediately removed, an opening made through the apex of the fang, a weak solution of nitrate of silver injected, going through and coming out where the matter was discharging; and creosote with floss silk introduced in the new cavity. This was repeated about once a week for three or four months, when the mouth had become quite healthy, and the tooth sufficiently well to fill. He then introduced a platinum pin into the fang, letting it extend down so as to form the corner of the tooth which had been broken off. Gold was then packed around this pin in such a manner as to make a solid filling from the apex to the cutting edge of the tooth, building out and forming it the same as it was before being broken. Six months after, he met the patient, and asked how the tooth got along? The reply was, I can bite a wire in two with that tooth.

Dr. Fuller (of N. H.) obtained leave to introduce the following resolution:

*Resolved*, That the Treasurer be directed to pay over to Dr. T. Potter, of Norwich, Ct., out of any funds that may remain in his hands after the expenses of this Convention are paid, the sum of one hundred and fifty-four dollars and twenty cents; that being the balance due to him for expenses incurred in defence of the air chamber patent suit, with Gilbert, of New Haven.

Dr. Rogers hoped the Convention would vote for it, as this Gilbert was going around the country and threatening to sue dentists, and Dr. Potter had done a service to the profession in carrying the suit up and having it decided in his favor; now we shall be exempt from his visitations. He commended his honesty in refusing to compromise the suit, and urged the justness of aiding him in the defence.

The same view was taken by Drs. Atkinson, Asay and others, and the resolution passed unanimously.

Dr. Fuller, of N. H., had varied his filling during the last year. Thought the undercut or shoulder system dangerous, apt to find the teeth in bad condition. Uses the gouges of Dr. Forbes; by these he can cut away the fissure, and get the orifice he wants. Where the cavity is deep and the nerve is not exposed and a solid filling is needed, has the cavity and gold cone shaped. It thus has the principle of the wedge, and he can put it down firmer.

Dr. Bristol, of Danville, does not, as a general thing, find filth in cases of undercuts. Wants the least possible amount of gold to press in filling. Thought it difficult to condense the plug if the cylinder filled the cavity fully, as proposed by Dr. Forbes.

Dr. Forbes puts in as large a cylinder as the cavity will bear, and for filling uses a flat instrument serrated. Packs from the side backwards,

Dr. Taylor. If he has undercuts fills them first. Suggested that the condensation acts not so much against the ends, as they are parallel planes coming straight together, and do not require that amount of pressure.

Dr. Barker said very few remarks had been made on difficult cases, and asked Dr. Atkinson how he would fill spoon shaped cavities that were sensitive.

Dr. Atkinson spoke of the use of the mallet. When the tooth is in a state of inflammation he cleans it to the point of the fang. Often, if abscesses have formed and are discharging, can fill same day. When the cavity is open he prefers a dressing that is tenacious, that more of its particles may be attached to the tooth. Uses saddler's silk. Prefers creosote and iodine in inflammation. It is well known that nothing is so good for compressing any substance as frequent blows of a mallet. Has used this instrument a great many years. Has succeeded in filling teeth that it was difficult to graduate the force in any other way so well. Can generally graduate the force by the mallet better than by the hand. Uses small serrated pluggers. Is not confined to any gold, but generally uses No. 3. Commences to use the mallet from the time the gold is put in. Can be best used on the molars. His mallet is three inches long, handle about twelve inches. Had used the one he now has for twenty years.

Dr. Fuller inquired if Dr. A. used the mallet with both hands.

Dr. Atkinson replied, that he had not become reduced to that physical necessity yet. (Laughter.) Uses cotton and creosote to seal the fang, leaving it there for ever. Conceives the benefits of creosote to be, it stimulates an effusion of coagulable lymph.

Dr. Watt applies creosote. Has no objection to leaving the cotton in. Thinks the carbonate of albumen is formed in the tooth by its use.

Dr. Chase related a case where a pledget of cotton, saturated with creosote, had remained in a cavity for six years, at the end of which time it was taken out and the tooth found well preserved.

A number of places were proposed for the next meeting of the Convention. Dr. Westcott named Syracuse, Dr. Atkinson, Cleveland; Detroit, Baltimore, Saratoga Springs, Chicago, and West Point, were proposed by others. Dr. Whitney proposed Mammoth Cave. Dr. Westcott thought it too great a cavity for the dentists to fill. Others asked if it was the intention to have the Convention cave in, &c. After almost adopting Dr. Westcott's proposition to meet at Syracuse, it was finally, after much voting, agreed to hold the next meeting at Saratoga Springs, the first Monday of August, 1860.

---

Friday, Fourth day.—The Convention took up "Mechanical Dentistry."

Dr. Miller made some remarks on electricity. This gentleman did not express much confidence in it as a local anæsthetic, remarking that with some patients it worked well, and again it was of no benefit. This seemed to be the sense of others. Showed a contrivance for clasping the tooth, so that the current could be applied directly to it, thus avoiding the necessity of passing a current through the body, or the liability of the patient to interfere to break the connection.

Dr. Franklin thought too little time was devoted by the Convention to this branch. The committee on programme had given an inferior position to it, when it was of vast importance, as thousands of dollars were invested in it every year; proposed that in future it be taken up at an earlier day. Advocated the use of the continuous gum work, which he said when made of vulcanite base combined all that was necessary to secure the best dentures the world has ever seen.

Dr. Dunn gave several cases where he had used porcelain plate, teeth and gum. This is a patented process, and he thinks it superior to any.

Dr. Loomis said that three years ago he had stated in Convention, that he had constructed an artificial denture entire of mineral paste. During the three years past, had used it extensively, and patients have always expressed satisfaction with it. These teeth will not wear out, need no repairing, &c. Could not think of any objection to them, unless it might be the fact that they were artificial. Would match it with any thing yet known. Did not find any difficulty in obtaining atmospheric pressure. It requires care in manipulation. He predicted its universal adoption, and urged dentists present to try it.

Dr. Fuller, of Peeksville, said we often have difficulty in obtaining a perfect articulation. Had done in this way, made his upper set first, then puts bicuspids on lower plate, and solders, using them for a guide for the adaptation of the molars and incisors. Did not have to grind, or otherwise disfigure them to bring the teeth on a level.

Dr. Allen offered some remarks on continuous gum work. Had given much attention to it, and thought the profession wanted light on it. It had stood the test for more than five years. Can be easily repaired. Almost impossible to break it in the mouth. If a tooth is broken by a fall or otherwise, it is ground to the gum, a new tooth fitted in, and the heat of the furnace consolidates it. If you have a perfect fit the patient is not conscious whether it weighs one penny-weight or one ounce, it will take many pounds to remove it. The teeth should be arranged so that there shall be no tipping; the pressure should come on the alveolar ridge, on the inner rather than on the outer side. Another advantage is, the dentist is enabled to select such an expression as he needs. Must study the case as the artist does his picture.

Dr. Goldy remarked, that he wore a set of the continuous gum work. The only objection that occurred to him was, it had produced considerable absorption of the gum.

Dr. Allen said that an experience of thirty years had convinced him that absorption took place, no matter what plate was used. He had not seen any cases where the additional weight had produced absorption.

Dr. Roberts asked if pressure produced absorption of the alveolar process. He had made a continuous gum plate, that weighed four ounces, for a gentleman who has worn it five years, and no absorption is apparent. He would not part with it for any money.

Dr. Butler thought absorption was most hastened by wearing temporary sets, and that it even occurs without any.

Dr. Roberts related the case of a lady, in whose lower jaw he had fitted a temporary plate of silver three years ago. He had fitted several sets since, that become loose in a few months. Absorption still continues—the patient now wants a new plate, and he asked what he should do to overcome the difficulty.

Dr. Chase asked Dr. Allen how he overcomes the contraction of the centre in his plate.

Dr. A. replied, after first baking, he brings the teeth to the cast, no matter if it does crack some at the heel; never remembers a case where the cracking has been of material injury.

Dr. Chase explained how he overcome the shrinkage of the metallic die. He trims out the centre of the palatine arch each side of the median line. Also overcomes the contraction caused by baking the continuous gum, in the same manner.

Dr. Allen said, that tin, zinc and bismuth make a fusible cast, in which there is less shrinkage than in any other. Would like to hear from Dr. Franklin on the subject of casts.

Dr. Franklin. Plaster after an hour, begins to expand, and continues to for five hours. If put in a bottle after being wet up it bursts it. He never wets it up before he wants to use it. His plan in melting zinc was to watch it—never allows all in the ladle to become fluid—stirs it constantly, to prevent crystallization, till nearly cold. Does not use it more than four times. Did not think the expansion of plaster could be overcome; it is about one-sixteenth of an inch in an ordinary cast. Bakes his teeth on continuous gum, on the cutting edge.

The Convention then passed to the subject of "Professional Intercourse."

Dr. Wilson then read an essay on Quacks and Quackery.

The reading of this paper was frequently interrupted by applause, and on its completion, a resolution of thanks was moved and carried unanimously. Dr. Forbes and others wanted it published in pamphlet form, some members agreeing to take as high as 500 copies.

Dr. Taylor, of Cincinnati, then read the following paper:*

Dr. Fuller, of N. H., offered the following:

*Resolved,* That the thanks of this Convention be tendered to the late President, Dr. Isaiah Forbes, for the ability and impartiality with which he has presided over its deliberations; and also to his associate officers, Dr. Rogers, Vice-President, Dr. Taft, Recording Secretary, Dr. Chase, Corresponding Secretary, and Dr. Roberts, Treasurer, for the careful attention which has characterised the performance of their several duties.

*Resolved,* That the thanks of this Convention be tendered to Dr. L. W. Rogers, for the faithful manner in which he has discharged his duties as a committee to arrange preliminaries for the holding of the present Convention, and that he be appointed a committee to make like arrangements for the Convention at Saratoga Springs, in 1860. Carried.

Dr. Taft read the following letter.

CROWN POINT, *March*, 1859.

About the last of March, an Irish woman came into my office and requested me to examine her mouth. I did so, and found, to my great astonishment, something I had

* The paper referred to has not yet been received—EDS.

never seen before. I examined it thoroughly, and pronounced it a cancerous tumor. It was situated on the left side of the lower jaw. I found the fangs of the second molar remaining, and removed them with considerable dfficulty; a large quantity of pus followed. I prepared a wash for cleansing her mouth, and told her to call in a few days. In a fortnight after the time, by the assistance of Dr. Doolittle, I removed the tumor after considerable difficulty, as it was attached to the alveolar ridge very firmly. We also had to remove the first molar.

July 30, 1859. J. W. H. Tefft, *Dentist.*

Dr. Perine showed a very interesting specimen of antique English teeth. It is an upper denture, made of sea horse base, ingeniously carved to fit the gum. The teeth themselves were taken from the jaw of a calf, and are pivoted with gold to the base. These teeth were made in England about sixty years ago, and cost £30. The same set were duplicated by Dr. Sizer, of Ct., (now deceased,) on a gold plate, at a cost of sixty dollars.

Dr. Forbes remarked, he had received a letter from a friend in St. Louis, who has been experimenting on oxyd of zinc for filling teeth. A new preparation of white filling—he is anxious to have dentists try it. The receipt consists of—

No. 1.—Calcined oxyd of zinc, 9 parts.
Finely powdered borax, 1 part.
" " silex, 2 parts.
All mixed well together.

No. 2.—Liquid—merely a concentrated solution of chloride of zinc, into which is to be dropped the smallest quantity of silicate of soda, or soluble glass.

Make a stiff paste of the two—i. e. No. 1 and 2.

The Convention then adjourned.

